# Quantification of mitochondrial DNA mutation load

**DOI:** 10.1111/j.1474-9726.2009.00505.x

**Published:** 2009-10

**Authors:** Laura C Greaves, Nina E Beadle, Geoffrey A Taylor, Daniel Commane, John C Mathers, Konstantin Khrapko, Doug M Turnbull

**Affiliations:** 1 Mitochondrial Research Group, Institute for Ageing and Health, Medical School, University of Newcastle upon TyneFramlington Place, Newcastle upon Tyne, NE2 4HH, UK; 2 Human Nutrition Research Centre, Institute for Ageing and Health, Newcastle UniversityFramlington Place, Newcastle upon Tyne, NE2 4HH, UK; 3 Beth Israel Deaconess Medical Center330 Brookline Avenue, Boston, MA 02215, USA

**Keywords:** ageing, cloning, colon, human, mitochondria, mitochondrial DNA, mutation load, polymerase chain reaction

## Abstract

Mitochondrial DNA (mtDNA) mutations are an important cause of genetic disease and have been proposed to play a role in the ageing process. Quantification of total mtDNA mutation load in ageing tissues is difficult as mutational events are rare in a background of wild-type molecules, and detection of individual mutated molecules is beyond the sensitivity of most sequencing based techniques. The methods currently most commonly used to document the incidence of mtDNA point mutations in ageing include post-PCR cloning, single-molecule PCR and the random mutation capture assay. The mtDNA mutation load obtained by these different techniques varies by orders of magnitude, but direct comparison of the three techniques on the same ageing human tissue has not been performed. We assess the procedures and practicalities involved in each of these three assays and discuss the results obtained by investigation of mutation loads in colonic mucosal biopsies from ten human subjects.

## Introduction

Mitochondrial DNA (mtDNA) is the only extrachromosomal source of DNA in animal cells (Nass, 1966). In humans, it is a ∼16.6 kb double stranded molecule containing 13 protein encoding genes which are all essential subunits of the mitochondrial oxidative phosphorylation system, as well as 22 tRNAs and two rRNAs which enable mitochondria to synthesise some of their own proteins (Anderson *et al.*, 1981). Mutations in mtDNA, both point mutations and large scale rearrangements, are an important cause of genetic disease (Taylor & Turnbull, 2005), and they have also been proposed to play a role in the ageing process (Miquel *et al.*, 1980).

Mitochondrial DNA mutations have been shown to accumulate with age in a number of postmitotic [e.g. brain (Cottrell *et al.*, 2001a), heart (Cortopassi & Arnheim, 1990), skeletal muscle (Cooper *et al.*, 1992)] and mitotic [colonic (Taylor *et al.*, 2003) and buccal epithelium (Nekhaeva *et al.*, 2002)] human tissues. These mutations have been shown to clonally expand to a level, which causes respiratory chain deficiency. These respiratory chain deficient cells can be detected using histochemical techniques (Old & Johnson, 1989) and fractions of respiratory chain deficient cells calculated with ease. However, quantifying the total mtDNA mutation load in ageing tissues is challenging but of fundamental importance if we are to understand the importance of mtDNA defects in human ageing.

A number of previous studies in human brain (Simon *et al.*, 2001) skeletal muscle (Del Bo *et al.*, 2002) and blood (Wilding *et al.*, 2006), and numerous tissues in the mitochondrial mutator mouse (Trifunovic *et al.*, 2004; Kujoth *et al.*, 2005) have employed a post-PCR cloning strategy. This technique involves DNA extraction followed by PCR amplification of a target sequence in the mitochondrial genome with a high-fidelity DNA polymerase. These PCR products are then cloned in a bacterial vector, and the plasmid DNA re-amplified and sequenced. Each individual PCR product is a copy of one mtDNA molecule and by sequencing a large number of clones; the mutation load per base pair can be quantified. This technique has come under criticism recently as all DNA polymerase enzymes, including high fidelity enzymes, have an intrinsic error rate. Upon cloning, polymerase errors cannot be distinguished from true mutations and may give an overestimation of mutation load. This can be controlled for by ‘cloning a clone’ to calculate the polymerase error rate, which can then be subtracted from the observed mutation frequency. This will not, however, control for the artefactual fixation of base adducts into mutations, which is also thought to occur with these enzymes (Kraytsberg *et al.*, 2008). An alternative method is single-molecule PCR (Kraytsberg & Khrapko, 2005). This involves extraction of mtDNA from tissue sections followed by serial dilution of the DNA down to a dilution in which only one in every five PCR reactions contains an amplifiable mtDNA molecule. This ensures that only one molecule is being studied. This method allows any polymerase errors to be identified. Indeed, any nascent PCR strand that acquires a polymerase error necessarily co-amplifies with error-free fragments, and thus will appear on the electropherogram as a heteroplasmic peak and will be excluded from analysis. Single molecule PCR (smPCR) may or may not exclude base adducts converted to mutations, depending on DNA quality (Kraytsberg *et al.*, 2008). A third method has recently been developed, the random mutation capture (RMC) assay (Vermulst *et al.*, 2007). This assay avoids the possibility of both polymerase errors and fixation of base adducts into mutations. The template DNA undergoes restriction digestion prior to PCR, so that all molecules which are wild-type at that restriction site will be cut and only molecules with a mutation in that site remain intact. PCR primers which flank the restriction site are then used to amplify the mutant molecules only. The starting template copy number is quantified using real-time PCR, and the mutation frequency calculated as the number of mutant bases per total number of base pairs screened. In view of the importance of studying total mtDNA point mutation load in ageing tissues, we have performed a systematic study to directly compare the three methods of quantifying mutation load to determine if there are differences in the results obtained with the different techniques. We also wished to assess any difficulties there may be in the set up of the techniques and their possible applications.

## Results

Our first objective was to assess the practicalities of setting up and performing each technique. Post-PCR cloning is a simple technique to establish as the whole process can be performed using a commercially available kit and an advantage of the post-PCR cloning technique is that it is able to be carried out on small amounts of tissue or individual cells, not feasible with the RMC assay. Single molecule PCR is also a relatively straight forward technique to establish; however, time must be taken to get the reaction conditions (primers, number of cycles, cycling conditions) right, and extra care must be taken to avoid cross-contamination. For a detailed review of these see Kraytsberg *et al.*, 2008 (Kraytsberg *et al.*, 2008). One disadvantage of this technique is the high dilutions of starting template required; to investigate point mutations it is necessary to dilute DNA so that only one in five wells contains an amplifiable template (Kraytsberg *et al.*, 2004). Therefore, to investigate high numbers of bases a large number of PCR reactions are required. In both techniques, post-cloning PCR and smPCR, amplification of nuclear pseudogenes is potentially possible as long as total DNA, rather than purified mtDNA, is used for analysis. Co-amplification of pseudogenes occurs only with certain combinations of PCR primers (Khrapko *et al.*, 1994) and it did not occur with the primers used in this work. Sequences of all nuclear pseudogenes are known (Woischnik & Moraes, 2002) and they are different from mtDNA in multiple nucleotide positions and are readily identifiable if they do co-amplify.

**Fig. 1:**
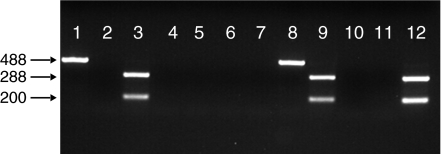
Ethidium bromide stained agarose gel image following random mutation capture analysis. Lanes 1 and 8: full length band following two rounds of digestion, this band would be excised and gel extracted for sequencing as it is presumed mutant. Lanes 3 and 9: PCR product which has cut during the second round of digestion with Taq1α. This represents a molecule which has not been digested during the first restriction digest and so has amplified during PCR. This shows the second round of digestion to be necessary so as to identify only true mutants, and avoid unnecessary sequencing. Lane 12: Control DNA from this subject which did not undergo digestion prior to PCR, but does digest after PCR, which shows that this subject does not have a polymorphic variant in the restriction site.

In our hands we found the RMC assay the most difficult to establish. The assay is extremely sensitive to primer sequence, concentration and degradation and it was especially important to check for amplification of nuclear pseudogenes. To avoid contamination we found it easier to amplify a larger product using a standard PCR protocol rather than real-time PCR, this meant that all products had to be electrophoresed following PCR, rather than only those products which give a positive signal on real-time PCR. There was also a problem with incomplete digestion of the mtDNA sample prior to PCR. We were able to overcome this by re-digesting the samples following PCR, then running the samples on an agarose gel. We then excised only the full length PCR products for sequencing. Figure 1 shows an agarose gel image with a mixture of full length mutant bands and wild-type bands, which have digested after PCR amplification. As a result of the changes in the protocol we wanted to assess the sensitivity and specificity of the RMC assay in our hands. To do this we cloned two PCR products in the region 5910–6396, one of which was wild-type at a Taq1α site within that region (6014–6017), one of which had a T>C transition at 6014. The PCR products were cloned using the method described above. Recombinant plasmids were identified and plasmid DNA was prepared using a plasmid mini-prep kit (Qiagen Ltd, Crawley, UK). DNA was then further purified using phenol/chloroform, ethanol precipitated, and resuspended in dH_2_O. DNA concentration was quantified by UV absorption at 260 nm and copy number calculated. Both the mutant and wild type clone were then diluted out so that each contained 1 × 10^6^ molecules of target sequence. The mutant clone was then diluted with the wild type clone in a ten-fold serial dilution from 10% mutant clone down to 0.00001% mutant clone. 1 μL of each dilution was then subjected to digestion with Taq1α for 10 h as described above in a total volume of 100 μL. This gave a total copy number in each sample of 10 000 per μL. Each dilution was then subjected to PCR as described above, the only difference being the primers used were L5912-L5932 and H6300-H6282. They were then re-digested and subjected to electrophoresis. We found that in a background of 10 000 copies of wild-type DNA the RMC assay could reliably identify one mutant molecule, and every PCR product that had been shown to be resistant to digestion by Taq1α following PCR contained the mutation within the Taq1α site. This showed that the RMC assay was both highly specific and highly sensitive. In this study we used a total of four different primer sets, one to quantitate copy number (H12360 and L12464), a set for the actual experiment (H6363 and L6851) and two different sets for the control experiments (H5912 and L6300, and H14838 and L15437). To check the efficiencies of the four primer sets, DNA from five different subjects was serially diluted from 1:10 to 1:10 000 and standard curves generated for each primer set from each subject using the real-time PCR conditions described in experimental procedures. The mean slopes of the curves were; H12360 and L12464:3.66 ± 0.08, H6363 and L6851:3.70 ± 016, H5912 and L6300:3.68 ± 0.09, and H14838 and L15437:3.67 ± 0.12. There was no significant difference in the primer efficiencies (one way anova, *P* = 0.88), therefore we were able to use them to make quantitative measurements.

**Fig. 2:**
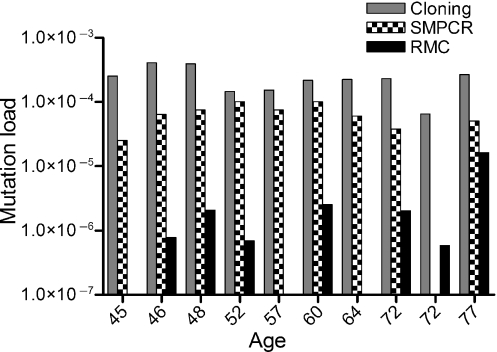
mtDNA mutation load in human colon with age. Quantification was by post-PCR cloning (grey bars), single molecule PCR (SMPCR, checked bars) and random mutation capture (RMC, black bars). Please note the logarithmic scale used on the *x*-axis to make the all of the data visible.

**Fig. 3:**
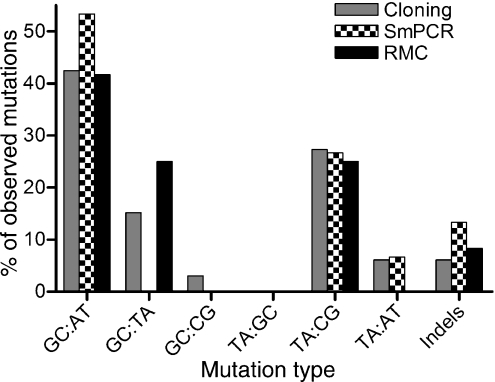
Types of mtDNA mutations observed in human colon. The types of mutations detected by post-PCR cloning (grey bars), single molecule PCR (SMPCR, checked bars) and random mutation capture (RMC, black bars) are shown. The data are expressed as the percentage of the total number of mutations observed by each technique.

Investigation of mtDNA derived from human colonic mucosa yielded average mutation loads of 2.3 × 10^−4^ ± 1.0 × 10^−4^ by PCR/cloning, 5.9 × 10^−5^ ± 3.2 × 10^−5^ by single molecule PCR, and 2.5 × 10^−6^ ± 4.9 × 10^−6^ for the RMC assay (mutation loads for individual subjects are in Table 1 and Table 2 shows the number of bases investigated). The differences in mutation loads were statistically significant (Friedman test *P*<0.0001). Interestingly none of the techniques showed any significant trend with age; however, this could be because of the small numbers of subjects involved in this study (Fig. 2). In all the three experiments the predominant type of mutation was a GA:CT transition (Table 3, Fig. 3), followed by TC:AG transitions. Transversions were quite rare except for GT:CA transversions by the RMC method. However, there were no statistically significant differences in the proportions of mutation types observed between any of the methods employed in this study (anova, *P* = 1.00). Interestingly, the type of mutations observed here are very similar to those which we have previously shown to be clonally expanded in cytochrome *c* oxidase deficient crypts in human colon (Taylor *et al.*, 2003; Greaves *et al.*, 2006). As a result of the short target of investigation by the RMC method we went on to look at a two additional Taq1α sites, 6014–6017 and 14596–14599, in the same 10 subjects as above and using an identical protocol. The mean mutation load at the 6014–6017 site was 7.91 × 10^−6^ ± 2.23 × 10^−5^, and the mean mutation load at the 14596–15499 site was 1.45 × 10^−6^ ± 2.23 × 10^−6^. There was no significant difference in the mutation loads observed at the three sites (Friedman test *P* = 0.36). The types of mutations observed at the three sites were also similar to those previously seen.

**Table 1:** mtDNA mutation loads observed in human colonic mucosa

		Mutation load (mutations per base pair)		
Subject	Age	PCR/Cloning	Single molecule PCR	RMC
1	45	2.5 × 10^−4^	2.5 × 10^−5^	0
2	46	4.1 × 10^−4^	6.4 × 10^−5^	7.7 × 10^−7^
3	48	3.9 × 10^−4^	7.5 × 10^−5^	2.1 × 10^−6^
4	52	1.5 × 10^−4^	1.0 × 10^−4^	6.9 × 10^−7^
5	57	1.5 × 10^−4^	7.6 × 10^−5^	0
6	60	2.2 × 10^−4^	1.0 × 10^−4^	2.5 × 10^−6^
7	64	2.2 × 10^−4^	6.1 × 10^−5^	0
8	72	2.3 × 10^−4^	3.8 × 10^−5^	2.0 × 10^−6^
9	72	6.5 × 10^−5^	0	5.8 × 10^−7^
10	77	2.7 × 10^−4^	5.1 × 10^−5^	1.6 × 10^−5^
Average ± SD		2.3 × 10^−4^ ± 1.0 × 10^−4^	5.9 × 10^−5^ ± 3.2 × 10^−5^	2.5 × 10^−6^ ± 4.9 × 10^−6^

**Table:** Number of bases investigated by each method

	No. bases investigated		
Subject	PCR/ Cloning	Single molecule PCR	RMC
1	11 842	39 600	1 360 432
2	12 224	31 100	1 282 481
3	15 280	26 400	958 437
4	13 752	19 800	1 435 947
5	12 988	26 400	799 051
6	13 752	19 800	794 205
7	13 370	33 000	703 448
8	12 988	26 400	491 479
9	15 280	19 800	5 135 368
10	14 898	19 800	122 879

**Table 3:** Mutations observed in human colonic mucosa

	Mutations detected		
Subject	PCR/Cloning	Single molecule PCR	RMC
1	m.16172 T>C, m.16254 A>T, m.16399A>G	m.14137C>T	None
2	m.1607A>G, m.16192 C>A, m.16244G>A, m.16327C>T, m.16111C>T	m.14182T>A, m.16060G>A	6564G>A
3	m.16069C>T, m.16126T>C, m.16174C>C, m.16217T>C, m.16293C>G, m.16311T>C	m.12448T>C, m.12731T>C	None
4	m.16216C>A, m.16389G>T	m.13769-13787 Del, 15984T>C	m.6565A>G
5	m.16125G>T, m.16223C>T	m.368A>G, m.15246G>A	m.6563C>T, m.6564G>T
6	m.16251C>T, m.16204G>A, m.16193 C ins, m.16193 2C ins	m.14167C>T, m.14419C>T	m.6363C>A, m.6564-6566 Del
7	m.16071C>T, m.16265A>G, m.16394C>A	m.13237 A Del, m.12301G>A	None
8	m.16070A>G, m.16318A>G, m.16184C>T	m.12991G>A	m.6565A>G
9	m.16059A>T	None	m.6564G>A, m.6564G>T, m.6562T>C
10	m.16071C>T, m.16104C>T, m.16114C>T, m.16192C>T	m.103G>A	m.6564G>A, m.6564G>A

## Discussion

An important goal in ageing research is to determine the incidence of mtDNA point mutations in ageing human tissues. To this effect we wanted to study the potential value of the three main methods currently available in colonic mucosa from 10 human subjects. There was a significantly lower mutation load detected by the RMC and single molecule PCR assays, than post-PCR cloning. In addition, there was also a difference in mutation load detected between the RMC assay and single molecule PCR. The differences between the three methods covered two orders of magnitude. There are many possible causes for these observed differences. PCR errors introduced during PCR before cloning could artificially increase the mutation load. There have been previous studies which have estimated the PCR error rate using high fidelity PCR enzymes. These estimates range from 1.4 × 10^−5^ (Smigrodzki *et al.*, 2004) to 2.65 × 10^−5^ (Wilding *et al.*, 2006) mutations per base pair. These estimations are comparable to or higher than the mutation loads we observe with smPCR and RMC and may interfere with mutational analysis, even when making comparative measurements. In addition to this, analysis of mutation types is not possible with cloning as there will be no way of differentiating between genuine and artificial mutations. Neither smPCR nor RMC are affected by these issues. A second potential source of artificially induced mutation in both cloning and smPCR is fixation of oxidative and/or other chemical adducts on the mtDNA molecules into mutations during PCR. Because of the restriction digest step prior to PCR, the RMC assay is not affected by these issues.

One advantage of the cloning and the single molecule PCR techniques is that the mutation load is calculated based on the exact number of base pairs sequenced, which can be simply counted; however, the RMC assay is reliant upon quantitation of exact starting copy number prior to digestion with Taq1α, which is dependent upon accurate quantification of DNA concentrations. A second disadvantage of the RMC assay is the short target of investigation; the short four base pair target of the restriction enzyme may not accurately reflect the mutation load across the whole genome. Indeed, there is evidence to suggest that RMC may underestimate mutant fraction. We have previously studied the incidence of clonally expanded mutations in individual colonic crypts (Taylor *et al.*, 2003; Greaves *et al.*, 2006). Estimates of clonal mutations are not subject to the various artefacts that plague PCR-based methods (Kraytsberg *et al.*, 2007) and are free of the limitations of RMC. Crypt-by-crypt analysis yielded an estimate of 2 × 10^−5^ clonal mutations per base pair in patients over 70 years of age (Taylor *et al.*, 2003). If we look only at the subjects over 70 years who were investigated in this study, this is three times higher than the RMC estimate and is approximately the same as the single molecule PCR estimate. The fraction of clonally expanded mutations represents a lower estimate of total mutant fraction because in the tissue there also may be an unknown proportion of nonexpanded mutations, which are expected to be detected by all the three methods compared here, but not by the crypt-by-crypt analysis.

Interestingly, one possible reason for the lower estimates obtained here by RMC as compared to crypt-by-crypt analysis, may be that in our experiments RMC did not detect any significantly expanded point mutations of the kind that are detected by the crypt-by-crypt analysis. The latter mutations constitute about 80% of the mtDNA of a colonic crypt on average (Taylor *et al.*, 2003). As in this work there were 100–1000 crypts per sample (depending on the biopsy size), the fraction of such a clonally expanded mutation as quantified by RMC should be 2 × 10^−3^–2 × 10^−4^ [mutant fraction equals the fraction of mutations in a mutant crypt (0.8) divided by the number of crypts per sample (100–1000) and by the length of the target sequence (4 bp)]. The most abundant RMC-detected mutation frequency is at least 10 times less prominent than this (Table 1 sample 10), implying that the expansions detected by RMC are no larger than the equivalent of 1/10 of a fully mutant crypt. Of note, sample 10 (see Table 1) has a mutant fraction (as measured by RMC) at least 10-fold higher than that of all other samples, and this outlier is a major contributor to the apparent poor reproducibility/precision of RMC. Most likely in sample 10 there is a small (at most 1/10 of a fully mutant crypt) clonal expansion of a mutation that by chance mapped within the RMC target. This possibility is corroborated by the fact that both mutant molecules detected in this sample by RMC are identical, as expected for a clonal expansion. The above argument implies that a representative mutational assay based on RMC has to carefully consider the sample size. At low sample size RMC is expected to detect only nonexpanded mutations. At certain larger sample size (depending on mutant fraction and the exact size distribution of clonal expansions) RMC starts detecting individual clonal expansions, with resulting large variability between samples (this is what is apparently observed in our experiments). At even greater sample size, multiple clonal expansions will be detected in each sample, and their contributions will average out to produce more stable estimates of the overall mutant fraction, which now will include the share of clonal mutations. Note that the other two methods where target sequence is much larger (i.e. ∼100-fold), the same stability of estimates is expected at approximately 100-fold smaller sample size. In other words, RMC is not an approach of choice for small samples with expected clonal expansions.

There were no significant differences in the types of mutations observed in the three data sets, and they were all very similar to the types of mutations which have been shown to clonally expand to high levels in human colon (Taylor *et al.*, 2003; Greaves *et al.*, 2006). This suggests that there is no selection for particular mutations to clonally expand.

In conclusion we have shown that there is a very wide variation in mtDNA mutation load with a number of different techniques. The RMC assay gives much lower mutation frequency and although may not give a totally accurate estimate of mutation load it is likely to be particularly useful for making comparative measurements between a set of subjects (e.g. disease state, dietary intake, genotype, age). Any errors in copy number, potential mutational hotspots, or underestimation of mutational load will be applicable to all subjects in the experiment. The RMC assay is not affected by PCR errors, is high-throughput, and is relatively cost-effective. However, because of the requirement of a mitochondrial preparation and a large sample size this technique can only be used to look at homogenate tissues and cannot be used to investigate individual cells or small pools of the same cell type. Single molecule PCR, though less high through-put, is free of PCR enzyme induced errors and may be a better option for exact quantification of mutation load, or to look at very small amounts of tissue.

## Experimental procedures

### Subjects

Colonic mucosal samples were taken from subjects (*n*= 10, age range 45–77 years) undergoing colonoscopy for a disturbance of bowel function in whom no evidence of neoplasia or other pathology was identified (BORICC 1 Study). Ethical approval was obtained by the Joint Ethics Committee of Newcastle and North Tyneside Health Authority and Northumbria NHS Trust Local Research Ethics Committee.

### Post-PCR cloning

To look at mutation load by PCR cloning, DNA was extracted from sections of fresh-frozen colonic mucosa using a standard lysis buffer (50 mm Tris–HCl pH 8.5, 1 mm EDTA, 0.5% Tween-20, 200 ng mL^−1^ proteinase K). 1 μL of DNA was used as template to amplify a 442 bp fragment containing the HVS-1 region of the mtDNA. PCR reactions comprised 30 pmol each of forward (L15995-L16014) and reverse (H16401-H16382) primer with 1 × PCR buffer (containing 2.0 mm magnesium sulphate), 200 μm dNTPs, 375 ng of DNA and 2.5 units of Pfu DNA polymerase (Stratagene, La Jolla, CA, USA). Amplification was carried out using a GeneAmp PCR System 9700 (Applied Biosystems, Warrington, UK) under the following conditions: 94 °C for 2 min followed by 30 cycles of 94 °C for 1 min, 52 °C for 1 min, 72 °C for 1 min and a final extension at 72 °C for 8 min. The PCR products were subjected to electrophoresis on a 1.5% agarose gel and the DNA purified using a QIAquick Gel Extraction Kit (Qiagen). A pCR-scriptTM Amp SK(+) cloning Kit (Stratagene) was used to clone the products following the manufacturer’s protocol. Recombinant plasmids were identified by blue–white colour selection. White clones were re-plated on ampicillin resistant plates and grown for ∼4 h. Colonies were lysed in 40 μL of 10% Triton X-100 and 1.3 μL of the Triton was then placed in a PCR reaction using the above PCR conditions except using Ampitaq Gold DNA polymerase/buffer (Applied Biosystems) and using a 10 min at 95 °C starting temperature. PCR products were cycle sequenced using ABI BigDye chemistries per standard manufacturer’s protocols and analysed on an ABI3100 genetic analyser (Applied Biosystems). Sequences obtained were compared with the revised Cambridge Reference Sequence (rCRS) and the homogenate sequence for that patient, using SeqScape software (Applied Biosystems). Mutation load was calculated for each patient by dividing the number of mutant bases by the total number of base pairs sequenced. In a control experiment we PCR amplified and cloned a clone using exactly the same protocol as described in above. Analysis of 100 clones of this clone did not reveal any mutations. In a second control experiment we took DNA from five of the subjects and amplified a region of the cytochrome *b* gene, (primers used; L14797-L14816 and H15238-H15309) to see if there were any differences in the mutation load between the two areas. PCR, cloning and sequencing was carried out as above. There was no significant difference between the mutation load found in the HVS-1 region to that found in the cytochrome *b* region (*t*-test, *P* = 0.26, data not shown).

### Single molecule PCR

To analyse mutation load by single molecule PCR whole sections of colonic mucosa were lysed in 10 μL lysis buffer (10 mm EDTA, 0.5% SDS, 1% proteinase K) for 1 h at 37 °C. The DNA was then serially diluted in Tris–EDTA so that there was only one amplifiable molecule in every five wells of a PCR plate to ensure that we were looking at single molecules. Typically the optimal concentration was 1/1 000 000. PCR was performed using TaKaRa ExTak PCR system (Lonza Biologics plc, Wokingham, UK) according to manufacturer’s recommended conditions except that quadruple the volume of ExTak was used. After 40 cycles of first round PCR (95 °C for 20 s, 68 °C for 7 min), 3 μl of second round PCR mixture was added directly to the first round products and 25 further cycles of PCR carried out. First round primers were L10161-L10192 and H780-H744; second round primers were L10195-L10225 and H726-H694. PCR products were subjected to electrophoresis in a 0.8% agarose gel. All PCR products obtained were sequenced as above. Mutation load was calculated by dividing the number of mutant bases by the total number of base pairs sequenced.

### Random mutation capture

To study mutation load using the RMC assay tissue homogenization of the mucosal biopsy was carried out followed by differential centrifugation to obtain a crude mitochondrial pellet. Pellets were incubated in a buffer containing proteinase K, and mtDNA was subsequently extracted using phenol–chloroform followed by ethanol precipitation and resuspension in dH_2_O. mtDNA was then drop-dialysed using membrane filters (0.025 μm, Millipore (UK) Ltd, Watford, UK) to extract any excess salts. 1 μL of mtDNA was then digested with 100 U of Taq1α for 10 h with the addition of 100U every hour, giving a total volume of 100 μL. Absolute quantification of mtDNA copy number in the digested mtDNA sample was carried out using SYBRGreen real-time PCR on a Roche Lightcycler to a target template outside of a Taq1α restriction site between nucleotides 12360 and 12464 within the ND5 gene. Absolute quantitation was carried out by the standard curve method. A template was generated between nucleotides 12284 and 13005. This was then purified using a gel extraction kit (Qiagen) and quantified by UV absorption at 260 nm. It was then serially diluted and used to generate a standard curve. All measurements were made in triplicate. Having ascertained the copy number for each mtDNA sample the digested samples were diluted to no more than 5000 target bases per μL in dH_2_O. A Taq1α site within the gene for cytochrome *c* oxidase subunit 1 (6562–6565) was chosen for analysis. Each 25 μL PCR reaction contained 12.5 pmoles each of forward and reverse primer primer (L6363-L6381 and H6851-H6831) with 1 × PCR buffer (containing 2.0 mm magnesium sulphate), 200 μm dNTPs, and 1.25 U of Amplitaq Gold (Applied Biosystems). 1 μL of digested mtDNA was added to each PCR reaction. A minimum of 100 000 base pairs were analysed per patient, and the number of reactions carried out per patient was calculated depending on the mtDNA copy number in that sample. PCR conditions were as follows; 95 °C for 10 min followed by 55 cycles of 95 °C for 15 s, 57 °C for 15 s and 72 °C for 30 s, final extension was at 72 °C for 7 min. Following PCR each product was digested with 50 units of Taq1α for 1 h at 65 °C, followed by 10 min at 80 °C to inactivate the enzyme. All products were then subjected to electrophoresis on a 1.5% agarose gel for 1 h at 200 v. All full length (488 bp) products were excised from the gel and the DNA extracted using a gel extraction kit (Qiagen). These products were then sequenced as described above. Mutation load was calculated by dividing the number of confirmed mutants by the total number of base pairs investigated.
